# Bis[(1-methyl-1*H*-tetra­zol-5-yl)sulfan­yl]ethane

**DOI:** 10.1107/S1600536811021957

**Published:** 2011-06-18

**Authors:** Chun-Rong Li, Tao Chen, Zheng-Qiang Xia

**Affiliations:** a School of Environmental Science & Engineering, Chang’an University, Xi’an 710054, Shaanxi, People’s Republic of China; b College of Chemistry and Materials Science, Northwest University, Xi’an 710069, Shaanxi, People’s Republic of China

## Abstract

The title compound, C_6_H_10_N_8_S_2_, was prepared by the nucleophilic substitution reaction of 5-mercapto-1-methyl­tetra­zole and dichloro­ethane. In the crystal, the mol­ecule possesses an approximate non-crystallographic twofold symmetry axis. The crystal packing is stabilized by weak inter­molecular C—H⋯N and π–π inter­actions [centroid–centroid distances = 3.448 (6), 3.5085 (5) and 3.4591 (2) Å]. The two five-membered rings form a dihedral angle of 1.9 (2)°.

## Related literature

For the synthesis and structures of closely related compounds, see: She *et al.* (2006 ▶); Wei *et al.* (2011 ▶). For the pharmacological activity of tetra­zole-containing compounds, see: Gilchrist (1992 ▶); Armour *et al.* (1996 ▶); Upadhayaya *et al.* (2004 ▶). For applications of tetra­zole derivatives in coordination chemistry and as energetic materials, see: Zhao *et al.* (2008 ▶); Wang *et al.* (2009 ▶).
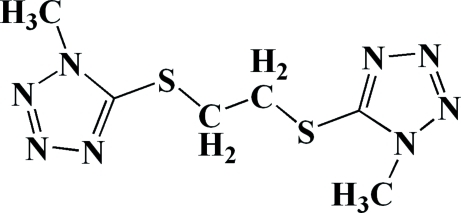


## Experimental

### 

#### Crystal data

C_6_H_10_N_8_S_2_
*M* *_r_* = 258.34
Triclinic, 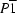

*a* = 7.5905 (17) Å
*b* = 7.9958 (17) Å
*c* = 10.398 (2) Å
α = 95.206 (3)°
β = 92.922 (3)°
γ = 115.109 (2)°
*V* = 566.3 (2) Å^3^
*Z* = 2
Mo *K*α radiation
μ = 0.46 mm^−1^
*T* = 296 K
0.31 × 0.27 × 0.04 mm

#### Data collection

Bruker APEXII CCD diffractometer
Absorption correction: multi-scan (*SADABS*; Bruker, 2002 ▶) *T*_min_ = 0.868, *T*_max_ = 0.982
2874 measured reflections
1972 independent reflections
1454 reflections with *I* > 2σ(*I*)
*R*_int_ = 0.017

#### Refinement

*R*[*F* ^2^ > 2σ(*F* ^2^)] = 0.047
*wR*(*F* ^2^) = 0.135
*S* = 1.39
1972 reflections
147 parameters
H-atom parameters constrained
Δρ_max_ = 0.26 e Å^−3^
Δρ_min_ = −0.26 e Å^−3^

### 

Data collection: *SMART* (Bruker, 2002 ▶); cell refinement: *SAINT* (Bruker, 2002 ▶); data reduction: *SAINT*; program(s) used to solve structure: *SHELXS97* (Sheldrick, 2008 ▶); program(s) used to refine structure: *SHELXL97* (Sheldrick, 2008 ▶); molecular graphics: *ORTEP-3* (Farrugia, 1997 ▶); software used to prepare material for publication: *SHELXL97*.

## Supplementary Material

Crystal structure: contains datablock(s) global, I. DOI: 10.1107/S1600536811021957/yk2010sup1.cif

Structure factors: contains datablock(s) I. DOI: 10.1107/S1600536811021957/yk2010Isup2.hkl

Supplementary material file. DOI: 10.1107/S1600536811021957/yk2010Isup3.cml

Additional supplementary materials:  crystallographic information; 3D view; checkCIF report

## Figures and Tables

**Table 1:** Hydrogen-bond geometry (Å, °)

*D*—H⋯*A*	*D*—H	H⋯*A*	*D*⋯*A*	*D*—H⋯*A*
C1—H1*A*⋯N4^i^	0.96	2.49	3.413 (5)	161
C6—H6*B*⋯N5^ii^	0.96	2.43	3.355 (5)	161

Symmetry codes: (i) 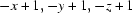; (ii) 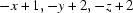.

**Fig. 1.**
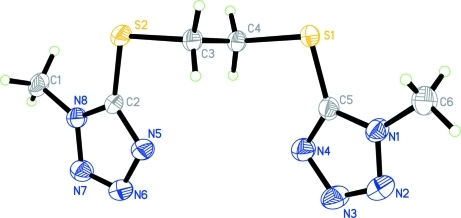
Molecular structure of the title compound, showing the atom labeling scheme and displacement ellipsoids drawn at the 30% probability level.

**Fig. 2.**
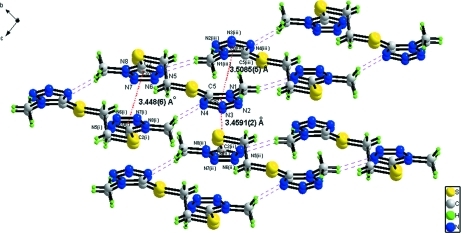
A view of the crystal packing of the title compound, showing the C–H···N and π-π interactions. Symmetry operators: i 1 - x, 1 - y, 1 - z; iix, 1 + y, z; iii 1 - x, 2 - y, x-z.

## supplementary crystallographic information

### Comment

As is well known, tetrazole-containing compounds are used in pharmaceutics, where they play a stimulative or sedative role for the central nervous system (Gilchrist, 1992; Armour *et al.*, 1996). Due to the various coordination modes of tetrazole group and high content of nitrogen, tetrazole derivatives have been widely applied in coordination chemistry (Zhao *et al.*, 2008) and the chemistry of energetic materials (Wang *et al.*, 2009). The title compound is a derivative of tetrazole. Nevertheless, reports on its use in pharmaceutical, coordination chemistry or energetic materials are very scarce. Here we report its crystal structure as a prerequisite of further investigation of possibilities for its use in the above fields.

In the crystal structure of the title, the molecule possesses approximate non-crystallographic twofold symmetry axis. Two 1-methyltetrazole groups are linked by —S—C_2_H_4_—S— bridge, and the two five-membered rings form a dihedral angle of 1.92° (Fig. 1). The values for the S1—C4 and S2—C3 bonds in the bridge [1.812 (4) Å and 1.814 (4) Å] are longer than the distances of S1—C5 and S2—C2 bonds [1.738 (3) Å and 1.725 (4) Å]. This difference can be attributed by electron attracting effect of 1-methyltetrazole groups. As shown in Fig. 2, tetrazole rings form stacks [intercentroid distances *Cg*1—*Cg*1(i) = 3.448 (6) Å, *Cg*2—*Cg*2(iii) = 3.5085 (5) Å, *Cg*1(ii)-*Cg*2 = 3.4591 (2) Å, (i) 1 - *x*, 1 - *y*, 1 - *z*; (ii) *x*, 1 + *y*, *z*; (iii) 1 - *x*, 2 - *y*, *x*-*z*].

### Experimental

Sodium hydroxide (1.7 g, 0.043 mol) was added to 5-mercapto-1-methyltetrazole (5 g, 0.043 mol) in dry dimethylsulfoxide (35 ml). The reaction mixture was stirred at 363 K for 1 h. Dichloroethane (3.1 ml, 0.0215 mol) was then added to the solution dropwise with the formation of a grey suspension. The suspension was stirred for 4 h, cooled to room temperature and filtered. The solvent was removed completely under reduced pressure, and the obtained residue was recrystallized from ethanol to give a colorless flaky crystalline product (2.94 g; m.p. 411 - 414 K).

### Refinement

All H atoms were positioned geometrically (C—H = 0.96 Å for CH_3_ and 0.97 Å for CH_2_ groups, respectively) and constrained to ride on their parent atoms with *U*_iso_(H) values set to 1.5 times *U*_eq_ of the parent atoms.

### Crystal data

**Table:**

C_6_H_10_N_8_S_2_	*Z* = 2
*M**_r_* = 258.34	*F*(000) = 268
Triclinic, *P*1	*D*_x_ = 1.515 Mg m^−^^3^*D*_m_ = 1.515 Mg m^−^^3^*D*_m_ measured by not measured
Hall symbol: -P 1	Mo *K*α radiation, λ = 0.71073 Å
*a* = 7.5905 (17) Å	Cell parameters from 816 reflections
*b* = 7.9958 (17) Å	θ = 3.0–24.3°
*c* = 10.398 (2) Å	µ = 0.46 mm^−^^1^
α = 95.206 (3)°	*T* = 296 K
β = 92.922 (3)°	Flake-like, colourless
γ = 115.109 (2)°	0.31 × 0.27 × 0.04 mm
*V* = 566.3 (2) Å^3^	

### Data collection

**Table:**

Bruker APEXII CCD diffractometer	1972 independent reflections
Radiation source: fine-focus sealed tube	1454 reflections with *I* > 2σ(*I*)
graphite	*R*_int_ = 0.017
φ and ω scans	θ_max_ = 25.1°, θ_min_ = 2.0°
Absorption correction: multi-scan (*SADABS*; Bruker, 2002)	*h* = −8→9
*T*_min_ = 0.868, *T*_max_ = 0.982	*k* = −9→9
2874 measured reflections	*l* = −12→12

### Refinement

**Table:**

Refinement on *F*^2^	Primary atom site location: structure-invariant direct methods
Least-squares matrix: full	Secondary atom site location: difference Fourier map
*R*[*F*^2^ > 2σ(*F*^2^)] = 0.047	Hydrogen site location: inferred from neighbouring sites
*wR*(*F*^2^) = 0.135	H-atom parameters constrained
*S* = 1.39	*w* = 1/[σ^2^(*F*_o_^2^) + (0.050*P*)^2^] where *P* = (*F*_o_^2^ + 2*F*_c_^2^)/3
1972 reflections	(Δ/σ)_max_ < 0.001
147 parameters	Δρ_max_ = 0.26 e Å^−^^3^
0 restraints	Δρ_min_ = −0.26 e Å^−^^3^

### Special details

**Table:**

Geometry. All e.s.d.'s (except the e.s.d. in the dihedral angle between two l.s. planes) are estimated using the full covariance matrix. The cell e.s.d.'s are taken into account individually in the estimation of e.s.d.'s in distances, angles and torsion angles; correlations between e.s.d.'s in cell parameters are only used when they are defined by crystal symmetry. An approximate (isotropic) treatment of cell e.s.d.'s is used for estimating e.s.d.'s involving l.s. planes.
Refinement. Refinement of *F*^2^ against ALL reflections. The weighted *R*-factor *wR* and goodness of fit *S* are based on *F*^2^, conventional *R*-factors *R* are based on *F*, with *F* set to zero for negative *F*^2^. The threshold expression of *F*^2^ > σ(*F*^2^) is used only for calculating *R*-factors(gt) *etc*. and is not relevant to the choice of reflections for refinement. *R*-factors based on *F*^2^ are statistically about twice as large as those based on *F*, and *R*- factors based on ALL data will be even larger.

### Fractional atomic coordinates and isotropic or equivalent isotropic displacement parameters (Å^2^)

**Table:**

	*x*	*y*	*z*	*U*_iso_*/*U*_eq_	
S1	0.09578 (12)	0.84390 (12)	0.77838 (9)	0.0521 (3)	
S2	0.09347 (12)	0.29485 (12)	0.68329 (10)	0.0556 (3)	
C3	0.1006 (5)	0.5022 (4)	0.7748 (3)	0.0475 (8)	
H3A	−0.0186	0.4695	0.8171	0.071*	
H3B	0.2096	0.5507	0.8418	0.071*	
C4	0.1213 (5)	0.6503 (4)	0.6906 (3)	0.0462 (8)	
H4A	0.0224	0.5975	0.6169	0.069*	
H4B	0.2485	0.6952	0.6576	0.069*	
N8	0.4054 (4)	0.2801 (4)	0.5763 (3)	0.0453 (7)	
N1	0.3945 (4)	1.1385 (4)	0.9194 (3)	0.0508 (7)	
N7	0.6021 (4)	0.3722 (4)	0.5857 (3)	0.0553 (8)	
C2	0.3378 (5)	0.3691 (4)	0.6618 (3)	0.0416 (8)	
N4	0.4956 (4)	0.9965 (4)	0.7756 (3)	0.0593 (8)	
N5	0.4887 (4)	0.5150 (4)	0.7245 (3)	0.0560 (8)	
N3	0.6501 (4)	1.1452 (5)	0.8422 (3)	0.0673 (9)	
C5	0.3392 (5)	0.9955 (5)	0.8249 (3)	0.0463 (8)	
N6	0.6500 (4)	0.5133 (4)	0.6750 (3)	0.0611 (8)	
C1	0.2987 (5)	0.1146 (5)	0.4834 (4)	0.0571 (10)	
H1A	0.3772	0.1141	0.4140	0.086*	
H1B	0.1791	0.1151	0.4489	0.086*	
H1C	0.2693	0.0055	0.5260	0.086*	
N2	0.5909 (5)	1.2321 (4)	0.9288 (3)	0.0664 (9)	
C6	0.2779 (6)	1.1957 (6)	1.0046 (4)	0.0694 (12)	
H6A	0.1958	1.2344	0.9540	0.104*	
H6B	0.3631	1.2973	1.0675	0.104*	
H6C	0.1981	1.0929	1.0482	0.104*	

### Atomic displacement parameters (Å^2^)

**Table:**

	*U*^11^	*U*^22^	*U*^33^	*U*^12^	*U*^13^	*U*^23^
S1	0.0445 (5)	0.0435 (5)	0.0671 (6)	0.0217 (4)	0.0033 (4)	−0.0113 (4)
S2	0.0421 (5)	0.0364 (5)	0.0800 (7)	0.0130 (4)	0.0051 (4)	−0.0121 (5)
C3	0.046 (2)	0.0407 (19)	0.054 (2)	0.0201 (16)	0.0055 (15)	−0.0083 (16)
C4	0.046 (2)	0.0393 (19)	0.0510 (19)	0.0189 (16)	0.0026 (15)	−0.0081 (16)
N8	0.0426 (16)	0.0439 (16)	0.0483 (16)	0.0188 (13)	0.0039 (13)	−0.0006 (13)
N1	0.0494 (18)	0.0460 (17)	0.0492 (16)	0.0150 (15)	0.0033 (13)	−0.0037 (14)
N7	0.0469 (18)	0.059 (2)	0.0606 (18)	0.0229 (16)	0.0091 (14)	0.0061 (16)
C2	0.0393 (18)	0.0323 (17)	0.0483 (18)	0.0128 (15)	−0.0012 (15)	−0.0021 (15)
N4	0.0489 (19)	0.057 (2)	0.067 (2)	0.0194 (16)	0.0076 (15)	−0.0027 (16)
N5	0.0457 (18)	0.0510 (18)	0.0647 (19)	0.0192 (15)	−0.0068 (15)	−0.0098 (15)
N3	0.0475 (19)	0.063 (2)	0.075 (2)	0.0088 (17)	0.0081 (17)	0.0056 (18)
C5	0.051 (2)	0.0419 (19)	0.0460 (19)	0.0220 (17)	−0.0010 (16)	−0.0004 (16)
N6	0.0456 (18)	0.055 (2)	0.074 (2)	0.0171 (15)	−0.0039 (16)	−0.0024 (17)
C1	0.064 (2)	0.0388 (19)	0.059 (2)	0.0167 (18)	0.0033 (18)	−0.0145 (17)
N2	0.057 (2)	0.057 (2)	0.069 (2)	0.0104 (17)	−0.0027 (17)	0.0026 (17)
C6	0.070 (3)	0.074 (3)	0.060 (2)	0.034 (2)	0.005 (2)	−0.023 (2)

### Geometric parameters (Å, °)

**Table:**

S1—C5	1.738 (4)	N1—C6	1.457 (4)
S1—C4	1.812 (3)	N7—N6	1.302 (4)
S2—C2	1.724 (3)	C2—N5	1.323 (4)
S2—C3	1.814 (3)	N4—C5	1.314 (4)
C3—C4	1.496 (5)	N4—N3	1.362 (4)
C3—H3A	0.9700	N5—N6	1.357 (4)
C3—H3B	0.9700	N3—N2	1.299 (4)
C4—H4A	0.9700	C1—H1A	0.9600
C4—H4B	0.9700	C1—H1B	0.9600
N8—C2	1.342 (4)	C1—H1C	0.9600
N8—N7	1.349 (4)	C6—H6A	0.9600
N8—C1	1.456 (4)	C6—H6B	0.9600
N1—C5	1.340 (4)	C6—H6C	0.9600
N1—N2	1.348 (4)		
			
C5—S1—C4	100.94 (15)	N5—C2—S2	128.0 (3)
C2—S2—C3	100.20 (15)	N8—C2—S2	123.6 (2)
C4—C3—S2	112.2 (2)	C5—N4—N3	105.8 (3)
C4—C3—H3A	109.2	C2—N5—N6	106.0 (3)
S2—C3—H3A	109.2	N2—N3—N4	110.7 (3)
C4—C3—H3B	109.2	N4—C5—N1	108.9 (3)
S2—C3—H3B	109.2	N4—C5—S1	128.2 (3)
H3A—C3—H3B	107.9	N1—C5—S1	122.9 (3)
C3—C4—S1	111.8 (2)	N7—N6—N5	110.8 (3)
C3—C4—H4A	109.3	N8—C1—H1A	109.5
S1—C4—H4A	109.3	N8—C1—H1B	109.5
C3—C4—H4B	109.3	H1A—C1—H1B	109.5
S1—C4—H4B	109.3	N8—C1—H1C	109.5
H4A—C4—H4B	107.9	H1A—C1—H1C	109.5
C2—N8—N7	108.6 (3)	H1B—C1—H1C	109.5
C2—N8—C1	129.7 (3)	N3—N2—N1	106.3 (3)
N7—N8—C1	121.7 (3)	N1—C6—H6A	109.5
C5—N1—N2	108.3 (3)	N1—C6—H6B	109.5
C5—N1—C6	130.2 (3)	H6A—C6—H6B	109.5
N2—N1—C6	121.5 (3)	N1—C6—H6C	109.5
N6—N7—N8	106.2 (2)	H6A—C6—H6C	109.5
N5—C2—N8	108.4 (3)	H6B—C6—H6C	109.5

### Hydrogen-bond geometry (Å, °)

**Table:**

*D*—H···*A*	*D*—H	H···*A*	*D*···*A*	*D*—H···*A*
C1—H1A···N4^i^	0.96	2.49	3.413 (5)	161
C6—H6B···N5^ii^	0.96	2.43	3.355 (5)	161

Symmetry codes: (i) −*x*+1, −*y*+1, −*z*+1; (ii) −*x*+1, −*y*+2, −*z*+2.
